# Correction: Shrewsbury, S.B. The Upper Nasal Space: Option for Systemic Drug Delivery, Mucosal Vaccines and “Nose-to-Brain”. *Pharmaceutics* 2023, *15*, 1720

**DOI:** 10.3390/pharmaceutics16060821

**Published:** 2024-06-18

**Authors:** Stephen B. Shrewsbury

**Affiliations:** Formerly of Impel Pharmaceuticals, 201 Elliott Ave West, Seattle, WA 98119, USA; steve.shrewsbury@me.com; Tel.: +1-(415)-250-1169

## Error in Table

In the original publication [1], there was a mistake in Table 1 as published. The corrected Table 1 appears below. Specifically, the SipNose device is intended to deliver both liquid and powder formulations to the upper nasal space, although clinical data are yet to be generated to demonstrate that.

## Text Correction

There was an error in the original publication. The bioavailability ascribed in the original publication to nasal midazolam was from a program developed by another company with another product (and device), not Nayzilam (as developed and marketed by UCB). The bioavailability for the approved (Nayzilam) product is less than that claimed by the competitor product and that has now been corrected in the text of Section 3.1. Lower Nasal Space (LNS) Delivery.

Section 2.1. Local Nasal and Sinus Disease

Many local sino-nasal diseases, specifically allergic rhinitis and rhinosinusitis with or without nasal polyps, have responded to the development of numerous topical corticosteroid sprays, starting with triamcinolone in 1957 but followed by beclomethasone, budesonide, flunisolide, fluticasone, mometasone and ciclesonide. All were delivered with either a pressurized metered-dose inhaler (pMDI) that was originally chlorofluorocarbon (CFC)-propelled but later changed to hydrofluoroalkane (HFA) or an aqueous product delivered by a “traditional” pump-spray.

Although many cases of chronic rhinosinusitis, especially with the additional pathology of nasal polyposis, are perennial, many patients have seasonal allergy problems making year-round administration of potent corticosteroids unnecessary. The target of the respiratory epithelium on the turbinates (conchae) of the LNS has been effectively reached by the traditional nasal spray delivering a diffuse cloud of droplets. In addition, systemic absorption is not desired, allowing for simpler formulations and delivery systems. Most recently, fluticasone has been approved for delivery by the OptiNose bidirectional Exhalation Delivery System (EDS) specifically for the treatment of chronic rhinosinusitis with nasal polyps (CRSwNP) [29] and is able to deliver more of the potent steroid “higher and deeper” into the nasal cavity (compared to traditional sprays) where many polyps originate [30]. The OptiNose device generally distributes better throughout the nasal cavity (than traditional nasal sprays) with its bidirectional design and is propelled by the patient’s own exhalation, which also elevates the vellum (soft palate), thus closing off the nose from the rest of the respiratory tract (Table 1). The nose piece directs the exhaled breath to push the drug product into one nostril, allowing it to reach the back of the nose, pass behind the septum and exit from the other nostril, hence creating the “bi-directional flow path” [16].

Section 3.1. Lower Nasal Space (LNS) Delivery

The delivery of drugs to the LNS can provide good bioavailability (BAV), i.e., access to the systemic circulation, with products such as Valtoco^®^ (diazepam) through the Aptar Unit Dose System (UDS) reported at 97% [72], although this good bioavailability was obtained with the help of dodecyl maltoside (DDM) as an absorption/permeation enhancer and Vitamin E to increase solubility. Another product with good nasal bioavailability is Nayzilam^®^ (midazolam), also delivered by Aptar’s UDS. Section 12.3 of the Prescribing Information states the absolute bioavailability to be approximately 44%. However, other intranasal midazolam formulations have been independently investigated [73], delivered by a different unit dose device obtained from Ing. Erich Pfeiffer GmbH, demonstrating better bioavailability ranging from 76 to 92%. These different formulations contained different concentrations of randomly methylated-β5-cyclodextrin (0, 2, 4, or 12%); different concentrations of saline and with or without 0.5% chitosan added as an absorption enhancer. In contrast, a very recent approval of Zavzpret^TM^ (vazegepant), also given by the Aptar UDS device [74], only reported ~5% bioavailability. The liquid formulation has the additional inactive ingredients: dextrose, hydrochloric acid, sodium hydroxide, succinic acid and water. In these examples, the same basic Aptar UDS delivery was used, presumably delivering to the same part of the nose (the LNS) with a diffuse nasal spray, showing that the addition of absorption enhancers was required to increase bioavailability. That, in turn, increases the complexity of the development program but does illustrate the extremes of the level of bioavailability that can be achieved through LNS delivery.

Section 3.2. Upper Nasal Space (UNS) Delivery

There is now a greater appreciation that drugs delivered to the upper nasal space (UNS) may experience faster and more extensive absorption than when delivered to the LNS. Data were initially generated with the OptiNose^®^ bi-directional system and then, more recently, by several clinical programs using the Precision Olfactory Delivery (or POD^®^) system (Table 1), which specifically targets the upper nasal space (UNS). These programs have served as important steps as they highlighted the complex nasal architecture (Figure 1a) and showed one of the potential benefits of delivering drugs to the olfactory epithelium lining in the UNS. As can be appreciated, a cloud or broad plume of droplets delivered into the nose by a mechanical pump will coat the surfaces of the septum, inferior turbinate and lateral wall, with some penetrating to the middle turbinate but little to the superior turbinate and even less to the upper nasal space lying mostly above the superior turbinate. The nose is designed to convey volatile molecules to the olfactory epithelium in the UNS carried by inhaled air but not to allow a broad plume of droplets/particles to reach it. The olfactory epithelium has distinct differences from the respiratory epithelium of the nose’s humidification and filtering system covering the three turbinates (or conchae), mostly located in the lower nasal space (Figure 1b). These differences lead to very different absorption profiles for drugs landing on different mucosae [75]. Few data have historically been generated looking at where nasal devices deliver their payloads, with options being to investigate in cadavers, nasal cavity replicas, nasal casts or by using in vivo gamma camera imaging [76].

Investigating the novel breath-actuated, bi-directional system, Djupesland and colleagues compared the systemic levels of midazolam with those obtained after traditional nasal spray delivery and IV delivery [77]. The 100 μL of bespoke formulation, including cyclodextrin, HPMC, EDTA and benzalkonium chloride, to 12 healthy adult subjects showed similar serum concentration curves for the two nasal devices, both with rapid T_max_ of 15 min and a geometric mean ratio (OptiMist/Traditional spray) of 97.6%, suggesting no difference in systemic delivery between the two devices. However, this group went on to deliver ^99m^Technetium-labelled aerosol [78] to nine healthy subjects and showed increased delivery to the UNS, of 32%, versus 11% with a traditional spray. Subsequent work with the OptiNose^®^ system reported 53.6% of the formulation reaching the UNS (upper and middle posterior regions) versus only 15.7% with the traditional liquid spray [79] with no lung deposition.

Hoekman and colleagues generated data supporting the differential delivery systems to the UNS with Precision Olfactory Delivery (POD^®^) using MAG-3 (^99m^Technetium-labelled peptide) as determined by SPECT imaging in seven healthy subjects [80]. For this assessment, the nasal cavity was divided into four sections (Figure 1c): (1) vestibule, (2) lower nasal space, (3) UNS and (4) nasopharynx, determined from MRI imaging [81]. The UNS is where the olfactory epithelium is confined, which will be further discussed in the context of N2B delivery below—although there is also an approximately equal area of respiratory epithelium in the UNS, as it covers the superior turbinate. POD delivered substantially more (approaching 50%) to the UNS and less to the vestibule than the traditional spray (Figure 1d). The olfactory epithelium covers ~5 cm^2^, representing ~3% of the total surface area of the nasal cavity [82]. The enhanced delivery of radioisotopes to the UNS then led to several clinical programs being launched with the POD system. The STOP 101 [83] clinical study specifically looked at the delivery of the same liquid formulation through an approved, traditional spray to the lower nasal space and compared it with POD (using the same device that was subsequently used in the phase 3 study [84] and thereafter approved and commercialized) delivering it to the UNS and showed a four-fold increase in (C_max_), an almost four-fold increase in absolute bioavailability (59% vs. 15%) and an almost three-fold increase in AUC_0-inf_ (Figure 2a). To accomplish this, the POD system has a novel design for the nozzle (dosing tip), which focuses the plume of emitted liquid droplets into a narrow plume [85], which is pictured in the publication. The differences in plume and aerosol characteristics between the two systems, with the same formulation of dihydroergotamine (DHE) mesylate, were further explained in a technical manuscript [86] as well as the Anderson Cascade Impactor data and using Spraytec technology, with a particle size distribution of Dv10 ranging from ~290 μm, Dv50 ranging from 402–472 μm across 10 samples from two lots and Dv90 ranging from 561–734 μm—all well above the respirable range. In addition, the novel nozzle of the POD device allows patients to position the device in the correct orientation if they follow the Instructions for Use and insert the device up to the “shoulder” of the actuator arm, which was confirmed to be easy to achieve in human factor testing.

To complete the clinical development for this INP104 program, the FDA required safety assessments of the UNS over 24 weeks with repeat dosing [84], which seemingly no other nasal delivery program had been asked to perform. This required the development of specific tools to assess the mucosal integrity of the olfactory epithelium and olfactory function, which detected no significant adverse effects over 24 and even 52 weeks [87].

A subsequent program, INP105, with a spray-dried powder formulation of olanzapine [88], showed in phase 1 a similar C_max_ and AUC as the same dose administered by intramuscular injection but with a faster T_max_; however with no approved IV formulation of olanzapine to compare against, absolute bioavailability could not be determined in this study (Figure 2b). The POD powder programs (both INP105 and INP103/107 with levodopa/carbidopa) [89] benefited from the previous development of both rodent and primate versions of the POD which expedited formulation development and pharmacokinetic characterization before, or even during, human trials [90], with the structure and function of the non-human primate nose being most similar to that of humans [91].

## References Correction

Additional references were included:

31. Unidose (UDS) Liquid Nasal Spray System. Available online: https://aptar.com/products/pharmaceutical/uds-unidose-liquid-nasal-spray-system/ (accessed on 9 June 2023).
32. Bidose (BDS) Liquid Nasal Spray System. Available online: https://aptar.com/products/pharmaceutical/bds-bidose-nasal-spray-system-manufacturer/ (accessed on 9 June 2023).
33. Unidose (UDS) Powder Nasal Spray System. Available online: https://aptar.com/products/pharmaceutical/unidose-nasal-powder-system-manufacturer/#:~:text=Aptar%20Pharma%E2%80%99s%20Unidose%20(UDS)%20Nasal,precise%20dose%20quickly%20and%20easily (accessed on 9 June 2023).
34. VP3 Multi-Dose Spray Pump. Available online: https://aptar.com/products/pharmaceutical/vp3-technology-platform/ (accessed on 9 June 2023).
35. Dihydroergotamine Mesylate. Available online: https://pi.bauschhealth.com/globalassets/BHC/PI/Migranal-PI.pdf (accessed on 9 June 2023).
36. Migranal. Available online: https://www.pdr.net/drug-summary/?drugLabelId=Migranal-dihydroergota-mine-mesylate-771 (accessed on 9 June 2023).
37. Flonase Allergy Relief Nasal Spray. Available online: https://www.flonase.com/products/flonase-allergy-relief/?gclid=CjwKCAi-Att2tBhBDEiwALZuhAEB-kI6eRoGWy2L-7zrmgp5zkSAX4ZcmpbvWvYIPwoRMvP0lijEufshoCvo8QAvD_BwE&gclsrc=aw.ds (accessed on 9 June 2023).
38. Trudhesa^®^. Available online: https://www.trudhesa.com/ (accessed on 9 June 2023).
39. Impel Pharmaceuticals. Available online: https://impelpharma.com/our-science/ (accessed on 9 June 2023).
40. INP105 Proof-of-concept Study for the Acute Treatment of Agitation in Adolescents and Young Adults With ASD (CALM 201). Available online: https://clinicaltrials.gov/study/NCT05163717?cond=Autism&term=NCT05163717&rank=1 (accessed on 9 June 2023).
41. The Future of Systemic and Central Nervous System (CNS) Therapies. Available online: https://kurvetx.com/ (accessed on 9 June 2023).
43. XHANCE^®^ (Fluticasone Propionate). Available online: https://www.optinose.com/products/xhance-fluticasone-propionate/ (accessed on 9 June 2023).
44. ONZETRA Xsail. Available online: https://www.onzetra.com/ (accessed on 9 June 2023).
45. STS101 (DHE Nasal Powder). Available online: https://www.satsumarx.com/our-research/sts101/ (accessed on 9 June 2023).
46. Aero Pump. Available online: https://www.aeropump.de/en/products/nasal (accessed on 9 June 2023).
47. Metered Pump Nasal Spray Bottles, 1 oz. Available online: https://pharmasystems.com/metered-pump-nasal-spray-bottles-1-oz-10271 (accessed on 9 June 2023).
48. Healthcare Mark II™. Available online: https://silgandispensing.com/products/mark-ii (accessed on 9 June 2023).
49. Nemera Ear, Nose, Throat. Available online: https://www.nemera.net/wp-content/uploads/2021/11/Nemera-ENT-brochure-digital.pdf (accessed on 9 June 2023).
51. SipNose Nasal Delivery System. Available online: https://sipnose.com/products/ (accessed on 9 June 2023).
52. MAD Nasal™ Intranasal Mucosal Atomization Device. Available online: https://www.teleflex.com/usa/en/product-areas/anesthesia/atomization/mad-nasal-device/ (accessed on 9 June 2023).
53. Teleflex Intranasal Drug Delivery. 2023. Available online: https://teleflex.com/usa/en/product-areas/emergency-medicine/intranasal-drug-delivery/mad-nasal-intranasal-device/index.html (accessed on 24 March 2023).
54. BD Accuspray™ Nasal Spray System. Available online: https://www.bd.com/en-us/products-and-solutions/products/product-families/accuspray-nasal-spray-system (accessed on 9 June 2023).
55. Becton Dickinson. BD AccusprayTM Nasal Spray System. 2023. Available online: https://drugdeliverysystems.bd.com/products/prefillable-syringe-systems/vaccine-syringes/accuspray-nasal-spray-system (accessed on 24 March 2023).
56. The Potential and the Challenges of Nasal Vaccination. Available online: https://www.ondrugdelivery.com/the-potential-and-the-challenges-of-nasal-vaccination/ (accessed on 9 June 2023).
57. Suman, J. Intranasal Vaccination: Rationale, Progress and Challenges. RDD Online 2023. In Proceedings of the RDD 2023, Antibes (Nice), France, 2–5 May 2023.

With this correction, the order of some references has been adjusted accordingly. The authors state that the scientific conclusions are unaffected. This correction was approved by the Academic Editor. The original publication has also been updated.

## Figures and Tables

**Table 1:** Examples of Different Nasal Delivery Systems (in development or approved). The bottom four devices are basically syringes with different nozzles attached so that as a Healthcare Practitioner pushes the plunger of the syringe in, the liquid vaccine is forced out of the various tips creating a local mist or broad plume of particles 30–100 μm in diameter (in the case of the MAD).

Manufacturer	Device	Propellant	No. Doses	[Drug/Other Programs]	Pros	Cons		Reference/ Website
Aptar (Crystal Lake, IL, USA) (Device only) Multiple programs with drug companies	Unidose (UDS) Liquid Nasal Spray	None No spring. Finger squeeze pressure.	1 of up to 100 µL	Zomig^®^ [zolmitriptan] Zavzpret^®^ [zavegepant] Valtoco^®^ [diazepam] Tosymra^®^ [DHE] [+ several others + multiple partnered programs	One-handed operation. Low actuation force. No priming. No shaking. Multiple approved FDA/EMA products—for migraine (and other indications).	No distinction between UNS and LNS deposition. Diffuse “cloud” of droplets/particles.		[31]
	Bidose (BDS) Liquid Nasal Spray	None Spring	2 × 100 µL	Spravato^®^ [esketamine]				[32]
	Unidose (UDS) Powder Nasal Spray	None No spring. Finger squeeze pressure	1	Baqsimi^®^ [glucagon]	Ideal for low solubility molecules, minimizes excipient requirements, allows for larger dose administration, improves bioavailability and enhances drug diffusion/absorption. No shaking/priming.	No distinction between UNS and LNS deposition. Complex formulation and may increase irritability.		[33]
	Multi-Dose (preservative-free) VP3 Nasal Spray Systems	None	Multiple	Tyrvaya^®^ [varenicline]	The “traditional” nasal spray for >40 years. Mechanical system. Reference pump for originator anti-allergy brands and their generic alternatives.	No distinction between UNS and LNS deposition.		[34]
Bausch Health (Laval, Canada) (Drug device combination product)	Traditional Nasal Spray	None	1	Migranal^®^ [DHE]	Approved (>20 years) for delivery of 2.0 mg liquid DHE for acute treatment of migraines. Has a “traditional” pump spray.	Requires assembly and priming. No distinction between UNS and LNS deposition. Diffuse “cloud” of droplets/particles.		[35,36]
Haleon (Weybridge, UK)	Sensimist (liquid)	None	60 doses	Flonase^®^ [fluticasone] (generics and OTC focus)	Now OTC product. Designed to deliver a cloud of liquid fluticasone droplets to the LNS for allergic rhinitis. Systemic absorption is NOT desired.	Typical systemic corticosteroid effects are seen with long-term use and absorption.		[37]
Impel (Seattle, WA, USA) (Drug-device combination products)	Precision Olfactory Delivery (POD^®^) (Liquid)	HFA134a	1	Trudhesa^®^ [DHE] INP102 [insulin]	Focus on UNS for N2B.Device adapted for each product. Approved for delivery of 1.45 mg liquid DHE formulation for acute treatment of migraine	First Generation device—requires assembly and priming.		[38]
	POD (Powder)	HFA134a	Multiple exchangeable tips	INP103 [levodopa] INP107 [carbidopa/levodopa combination]	Several iterations of the device; research (which uses drug powder products in capsules) and single (no assembly) or multiple (changeable tip) dose design	Although delivering to UNS, there are no data on N2B delivery.Remains “in development.”		[39]
	POD (Powder)	Nitrogen	1	INP105 [olanzapine]	Single-use, preloaded device (no priming or assembly)	Research and development halted Q1 2023 (for business reasons)		[40]
Kurve (Lynnwood, WA, USA) (Device only)	ViaNase^TM^ (liquid)	None	1	N/A [insulin]	Focus on UNS for N2B. Device adapted for each product. Sixteen programs, fourteen in-clinic. Battery powered.	Unreliable performance in large NIH AD-insulin trial		[41,42]
OptiNose (Yardley, PA, USA) Drug-device combination products	Xhance^®^ (OptiMist^®^) Bi-directional	None (breath powered)	120 sprays	Xhance^®^ [fluticasone]	Approved for CRSwNP. Broader dispersion around the nasal cavity. The soft palate closes during use preventing the drug from entering the pharynx (or lungs).	Patient unfamiliarity with blowing into own nose. Initial priming is needed, and shaking before every dose. Focus on nasal disease—not systemic or N2B delivery.		[43]
	Bi-directional Exhalation Delivery System (EDS)—Onzetra^®^ Xsail^®^		1	Onzetra^®^Xsail^®^ [sumatriptan]	More of the drug reaches UNS. Approved for the delivery of sumatriptan powder (for migraine).	Patient unfamiliarity with blowing into own nose.		[44]
Satsuma (Durham, NC, USA) (Drug-device combination product)	STS101—a manually squeezed plastic bottle	None	1	N/A [DHE]	Small, portable, and no assembly or priming, delivering 6.0 mg DHE powder	Two failed phase 3 studies, with device modifications in between to improve delivery. No distinction between UNS and LNS delivery		[45]
AeroPump (Hochheim am Main, Germany) (Device only)	AeroPump	None		N/A [insulin] (adaptable for use by OTC and generic drugs)	Requires priming. Simple. Easy to use.	Non-targeted, LNS delivery		[46]
PharmaSystems (Markham, ON, Canada) (Device only)	Metered Nasal Dispenser	None	Multiple	N/A [insulin] (Canadian pharmacy supplier)	Requires priming. Simple. Easy to use. Programs showed benefits in postoperative delirium and postoperative cognitive function	Non-targeted, LNS delivery		[47]
MeadWestvaco (Richmond, VA, USA)/Silgan (Richmond, VA, USA) (Device only)	Mistette MkII Pump	None	Single/Multiple?	N/A [insulin] (provide bottles/pumps for liquid formulation delivery)	Requires priming. Simple. Easy to use.	Non-targeted, LNS delivery.		[48]
Nemera (La Verpillière, France) (Device only)	SP270+	None	Single/Multiple	N/A [insulin]	Requires priming. Simple. Easy to use.	Program was discontinued due to variations in viscosity.		[49]
SipNose (Yokneam, Israel) (Drug-device combination products)	Sipnose	Compressed air	Single and Multiple dose	N/A [insulin] (seven programs; five in-clinic, three of their own, and two partnered)	Performed well in Patient-Centered Care Impact Analysis [50] compared to two invasive ROAs (intrathecal and intracerebroventricular injection. Liquid and powder formulations. Targeted for UNS delivery.		Clinical data awaited	[51]
Teleflex (Wayne, PA, USA) (Device only)	Mucosal Atomisation Device (MAD)	None	1	N/A [vaccines] (Astra Zeneca: ChAdOx1nCoV-19 vaccine)	Fitted to the tip of a standard syringe in clinical trials of vaccines		Liquid (vaccines or drugs) administration by HCP	[52,53]
BD (Franklin Lakes, NJ, USA) (Device only)	Accuspray^TM^	None	1	N/A [vaccines] 510(k) cleared	Fitted to the tip of a standard syringe in clinical trials of vaccines		Liquid (vaccines or drugs) administration by HCP	[54,55]
Aptar	BiVax	None	1 (either 200 μL or 500 μL—split across 2 nostrils)	N/A [vaccines]	The liquid vaccine can be transferred directly from vial to applicator.		Liquid (vaccines or drugs) administration by HCP	[56,57]
Aptar	LuerVax (nozzle only)	None	1	N/A [vaccines]	Fitted to the tip of a standard syringe in clinical trials of vaccines.		For HCP liquid administration	

AD = Alzheimer’s disease; CRSwNP = chronic rhinosinusitis with nasal polyps; DHE = dihydroergotamine (for migraine); EMA = European Medicines Agency; FDA = Food and Drug Administration; HCP = healthcare practitioner; HFA = hydrofluoroalkane; LNS = lower nasal space; N/A = not applicable (no approved product); N2B = nose-to-brain delivery; NIH = National Institutes of Health; OTC = over the counter; POD = Precision Olfactory Delivery; UNS = upper nasal space.
