# Supplementary material for: The Upper Nasal Space: Option for Systemic Drug Delivery, Mucosal Vaccines and “Nose-to-Brain”
Source: Pharmaceutics. 2023 Jun 13;15(6):1720. doi: 10.3390/pharmaceutics15061720 (PMC10303426; doi:10.3390/pharmaceutics15061720)
Supplement: Supplementary file 1 [file pharmaceutics-15-01720-s001.zip › pharmaceutics-2414525-supplementary.pdf]

Nasal Vaccines in Development (Supplementary Material)

Nasal delivery of vaccine was largely overlooked until relatively recently. The COVID-19 pandemic and the needs for rapid development, global distribution and administration of effective vaccine have changed that with many vaccine candidates now in development not only for SARS-CoV-2, the virus that causes COVID, but for other vital and even bacterial disease. And not only for those entering the body through the nose. A brief summary of how the viral antigens trigger immunity is provided here.

<u>Mucosal immunity</u>

The nasal epithelial M-cells move the antigen from apical to basal surface of the superficial epithelium where the underlying antigen presenting cells (APCs), dendritic cells or macrophages, are located. The APCs move to the regional lymph nodes and the Nasal Associate Lymphoid Tissue (NALT) where lymphoid tissue, T and B cells will process the presented antigen and develop both the mucosal IgA and the systemic IgG [112]. While B cells support the development and maintenance of memory T cells, these T cells are required for humoral response to protein or peptide antigen. Nearly 80% of total immunocytes are associated with mucosal surfaces in healthy adults [117]. In the human nose, lymphoid tissues, known as Waldeyer's ring, supports the lymphatic drainage from the oropharynx and nasopharynx although it is more diffuse than in many animal species [118]. Humans (and many other large mammals) do not have such well-defined NALT as rodents, but humans do have more defined tonsils – such as the paired pharyngeal tonsils (adenoids) and palatine tonsils ("tonsils") that are part of Waldeyer's ring along with the single pharyngeal and lingual tonsils [119]. In one postmortem study of 150 children who died before age 2 years, much of the NALT tissue identified was located around the middle turbinate (26%), with surprisingly more, (30%), around the upper nasal space [120]. There is no data on how or where the lymphoid tissue is organized in adults [120].

Recently, it has become recognized that mucosal immunity does not just confer immunity at the recipient nasal mucosa, but other mucosae, e.g., in the gut or urogenital tract, also develop immunity that may not be gained after systemic vaccine administration. This cross reactivity at distant mucosae is enabled by IgA whereas the systemic immunity conferred by IM (or subcutaneous (SC)) inoculation is conferred by IgG. The IgA mediated immune response is also faster – important when wishing to avoid the nasal mucosa becoming a reservoir for viral spread. Thus, nasal administration of a vaccine has the potential for much broader application than just to combat airborne viruses. Indeed, already Norovirus vaccine has been extensively investigated, using various virosomes (also called Virus Like Particles or VLPs) delivered by nasal spray, both as a monovalent vaccine and more recently a bivalent vaccine. But without the broad coverage against a wide diversity of the different strains (over 30 genotypes of Norovirus have so far been identified) that may give rise to acute gastroenteritis, an effective vaccine to protect against Noroviral disease remains elusive at this time [121].

<u>Vaccines</u>

Vaccines deliver viral antigen to the body to elicit immunity in one of 7 general systems **[Table S1]**: inactivated virus; live attenuated virus; viral vector; protein subunits; virus like particles; DNA or RNA based synthetic nucleotide-based vaccines. The immune response can

then be either dominated by cellular (T lymphocytes – especially CD8+ and CD4+) or humoral (antibodies – especially circulating IgG and IgA at mucosal surfaces) immunity – although that is an oversimplification.

In early 2020, reports of a new coronavirus, possibly spreading from a "wet market" in Wuhan, China began to emerge. This became the largest global pandemic since the 1918 "Spanish flu" but is not the only recent example of viral disease "jumping" to humans from an animal species. The H5N1 "bird flu" and the West African outbreak of Ebola were also both due to inter-species transfer from birds and bats respectively.
Three years later, SARS-CoV-2 is still with us and the virus, and its now numerous variants. However, the vaccines, several approved under Emergency Use Authorisation initially, have saved millions of lives. Their rapid development was in two cases based off newer RNA technology (from Moderna [122] and Pfizer-BioNTech [123]) following the initial viral genome being sequenced in January 2020 [124]. The more familiar vaccines based off viral vector vaccines, usually adenovirus, (Johnson & Johnson/Janssen [125]) or protein subunits (Novavax [126]), were also developed in a similar accelerated timeframe and while infection rates continue to surge, resolve and surge again, the rates of hospitalizations and deaths have remained much lower than before the various vaccines were launched.

Vaccination does not guarantee immunity and as the virus evolves, immunity becomes even less effective against new variants, especially if that immunity was against a specific protein (e.g., the spike, or S protein in the case of SARS-CoV-2) that may have changed as the different variants emerge. The hope is that newer "bivalent (booster) vaccine" inducing immunity against 2 different viral proteins may be more effective than the original monovalent vaccines, but that case has yet to be proven.

Both India (a 2-dose, primary inoculation, iNCOVACC®) and China (a booster dose delivered by nebulizer, Convidecia Air TR) have now approved nasal delivery for SARS-CoV-2 viral vector vaccines using adenovirus to deliver SARS-CoV-2 genetic material to nasal mucosal cells [127], although primary data from these two programs has not yet been published. Also unpublished are the data supporting Iran and Russia's nasal COVID vaccine programs although these vaccines are now in use in their respective countries. This contrasts with the more familiar IM (or SC) administration of vaccine where 67 of the currently approved 92 US vaccines are given by this route (as of January 2023). Adenoviral vector SARS-CoV-2 vaccine use was paused after the rare but serious complication of vaccine induced thrombocytopenia (VIT) [128] was reported in early 2021, although the exact mechanism causing this has yet to be fully elucidated.

Vaccine platform	Advantages	Disadvantages	Approved examples
Whole inactivated virus	Stronger immune response.<br>Safer than live attenuated virus	Epitope may be altered by inactivation process; Eosinophilic pulmonary pathology reported in animal models	Typhoid; Cholera; Hepatitis A; Influenza; Polio (Salk); SARS-CoV-2 (Sinovac; Wuhan Institute)

Live attenuated virus	Stronger immune response; Native antigen preserved; Mimics natural infection	Risk of reversion (to more virulent state) or residual virulence (safety risk – especially for immunocompromised)	Measles; Mumps; Polio (Sabin); Rotavirus; Yellow fever; BCG; Rubella; Varicella
Viral vector vaccine	Stronger immune response; Native antigen preserved; Mimics natural infection	More complex to manufacture; Risk of genomic integration; Pre-existing immunity against vector will dampen response	Ebola; SARS-CoV-2 (from Johnson & Johnson/Janssen; Astra Zeneca; Gamaleya; CanSino Biologics)
Subunit	Safe and well tolerated	Lower immunogenicity; Requires adjuvant or conjugate to increase response	Pertussis; Influenza; *Strep pneumonias; Hemophilus influenza*; SARS-CoV-2 (from Novavax)
Virus-like Particle (VLP)	Safe and well tolerated; Mimics native virus conformation	Lower immunogenicity; More complex to manufacture	Hepatitis B, Human Papillomavirus (HPV)
DNA	Can build once virus sequenced; Safe and well tolerated; Stable at room temperature; Highly adaptable to new pathogens; Native antigen expression	Lower immunogenicity; Difficult administration (intradermal electroporation); Risk of genomic integration, mutagenesis and oncogenesis	SARS-CoV-2 (from Zydus Cadila)<br>Several veterinary examples
RNA	Can build once virus sequenced; Safe and well tolerated; Highly adaptable to new pathogens; Native antigen expression; No genomic integration risk;	Lower immunogenicity; Requires cold storage and transportation; Risk of RNA-induced interferon response (cytokine storm)	SARS-CoV-2 (from Moderna; Pfizer/BioNTech)

Table S1 Different vaccine platforms (with some examples, advantages and disadvantages). Adapted from Li et al [129].

As interest in nasal vaccines has grown, so the Drug Delivery Systems (DDS) have become more sophisticated [130] – the actual pharmaceutical formulation intended to contain the viral antigen and, once in the body, deliver it to the target cells. With SARS-CoV-2, RNA vaccines have "come of age", and there are now programs delivering self-amplifying RNA

vaccines [131] which can be delivered in several sophisticated DDS, for instance polymeric and lipid nanoparticle (LNP) forms [37]. The early recognition that SARS-CoV-2 can lead to a loss of the sense of smell has perhaps increased the appreciation that protecting the nasal mucosa is an important public health and personal health goal.
These nasally delivered vaccine programs deposit on the respiratory mucosa of middle and inferior turbinates of the lower nasal space where the prolific mucus traps inhaled particles and microbes. The mucus with the trapped particle and microbes is then removed by rapid mucociliary clearance (MCC), with a turnover of ~21 minutes in humans [132,133].

Vaccine adjuvant
Material has long been added to vaccines to facilitate, encourage or exaggerate the immune response the human body mounts to the viral antigens. Only 3 of the 464 ongoing vaccine trials (March 2023) are purportedly “adjuvant free”. Aluminum salts have been added for over 70 years to vaccines for IM injection. Information about these salts and the many other adjuvants can be found on the Center for Disease Control website (https://www.cdc.gov/vaccinesafety/concerns/adjuvants.html), although several important vaccines, notably the RNA based COVID vaccines, do not include adjuvants. Some of these adjuvants have promising features extending beyond purely aiding the immune response. For instance, vaccine adjuvanted with AS04 had a unique in vitro droplet size distribution and when delivered to a nasal cast demonstrated a favorable deposition profile [37]. However, adjuvants can be harmful – as the Nasalflu vaccine which contained *Escherichia coli* heat-labile toxin demonstrated. The neurotoxin in the vaccine, available only in Switzerland in 2000, led to dozens of cases (at least 43) of Bell’s Palsy [132], which were thought to be caused by direct uptake of the toxin through ganglioside receptors into the olfactory bulb, (although contamination with Herpes simplex and an autoimmune reaction were also questioned) leading to the vaccine being withdrawn. However, 2 subsequent large population-based studies failed to confirm the connection [134,135]. Direct transmission of adjuvants or live-attenuated vaccine into the CNS may lead to unintended consequences, e.g., encephalitis and encephalopathy have been reported [136]. These unintended sequelae may support the speculation that delivery of some products to the nasal mucosa may indeed directly enter the CNS.

Excipients
Residence time for drug or vaccines once deposited on the nasal mucosa has to be long enough for the drug or antigens to be absorbed, leading to the common addition of either mucoadhesives or permeation enhancers, or sometimes both, as excipients to nasal formulations. Most mucoadhesives are polymers which on contact with the aqueous mucus become hydrated, swell and bind to the mucus, and can sometimes slow the otherwise brisk MCC [3].

Drug Delivery Systems (DDS)
For nasal delivery, 100 -200 μL is considered the optimal volume [111] and must contain the drug or vaccine, any adjuvant and any/all necessary excipients. Several novel DDS have generated promising data in humans to support nasal drug delivery: cyclopenta decalactone, chitosan, low methylated pectin and polyglycol mono- and diesters of hydroxystearate (70%) and polyethylene glycol (30%), and have been reviewed elsewhere [3] and also alkylsaccharides [111]. A more recent review looking at nasal delivery of

nanoformulations [20], highlighted the role of nanotechnology in the DDS for nasal delivery with polymeric nanosuspensions, nanogels or nanoliposomes; niosomes (highly biocompatible and biodegradable nanoscale vesicles); nanospheres and nanocapsules; polymeric nanomicelles; metal, gold, silver or magnetic nanoparticles and dendrimers all generating promising data. Some of these (e.g. leucine-5-encephalin or LENK) have been combined with an excipient (e.g. LENK has been combined with a chitosan-based nanoparticle) to generate a 5-fold increase in brain exposure [20]. Many of these technologies however are still looking for a device that can deliver their nanotechnology-assisted drug to the olfactory epithelium.

For vaccines, the DDS must also protect the antigen against the natural destructive enzymes in the mucus; support antigen recognition, presentation and immune response by vaccine alone or with any adjuvant; support vaccine delivery to the right compartments and to the appropriate cells and allow the vaccine enough time at the target site. Several current vaccines, as in the case of the approved nasal SARS-CoV-2 vaccines from India and China, use live but relatively harmless Adenovirus as the DDS. Adenoviral vectors, over 150 of which have been characterized, have been used for delivering not only viral genes to the host, but they have been used to deliver human genes for patients suffering from relevant genetic disease (usually a missing or mutated gene) in “gene therapy” [137]. The Adenoviral genes responsible for viral replication (E1 and/or E3) have been removed and replaced with the genes of interest, and the Adenovirus does not integrate into the host genome. In addition, the Adenovirus is capable of carrying a significant genetic payload, generates a strong immune response and has been used to carry viral (HIV, Ebola, Zika), bacteria (Tuberculosis), parasitic (Malaria) and cancer immunostimulatory material and is suitable for large scale bio-manufacturing without the resulting cold chain storage need of the RNA-based vaccines.
